# Effect of Amino Acid Infusion on Amniotic Fluid Index in Pregnancies Associated With Oligohydramnios and Fetal Growth Restriction

**DOI:** 10.7759/cureus.39027

**Published:** 2023-05-15

**Authors:** Akruti Shinde, Kamlesh Chaudhari, Deepika Dewani, Deepti Shrivastava

**Affiliations:** 1 Department of Obstetrics and Gynaecology, Jawaharlal Nehru Medical College, Datta Meghe Institute of Higher Education and Research, Wardha, IND

**Keywords:** amniotic fluid index, perinatal outcome, amino acid infusion, fetal growth restriction, oligohydramnios

## Abstract

Background

Oligohydramnios and fetal growth restriction have been known for ages, with increased risk of disease and death during antenatal, neonatal, and adult life leading to operative interventions and perinatal mortality and morbidity. The amniotic fluid index varies with gestational age and is used to detect fetal well-being. Various oral and IV hydration and amino acid infusion therapies are studied to improve amniotic fluid index (AFI) and fetal weight.

Objective

To study the effect of intravenous amino acid infusion on AFI in pregnancies associated with oligohydramnios and fetal growth restriction (FGR).

Material and methods

A semi-experimental study done in Acharya Vinoba Bhave Rural Hospital (AVBRH), Sawangi Meghe, Wardha enrolled pregnant women in the in-patient department (IPD) unit of Obstetrics & Gynecology and divided them into two groups of 52 each, which met inclusion and exclusion criteria. Group A received IV amino acid infusion on an alternate day, whereas group B received IV hydration, and serial monitoring was done till delivery.

Results

The mean gestational age at admission was 32.73 ± 2.21 in the IV amino acid group and 32.25 ± 2.27 in the IV hydration group. In both groups, the mean AFI at admission was observed at 4.93±2.03 cm and 4.22 ± 2.00 cm, respectively. The mean AFI on the 14th day in the IV amino acid group was 7.52 ± 2.04, and in the IV hydration group, 5.89± 2.20 with a significant p-value of <0.0001.

## Introduction

Amniotic fluid occupies the amniotic cavity surrounding the growing fetus during pregnancy. It is an alkaline, watery fluid that provides nutrition and protects the growing fetus. The protective effects of the amniotic fluid include its cushioning effect, which protects against mechanical and biological injury due to shear stresses and forces while creating a barrier against infection. The amniotic fluid maintains its homeostasis by regulating its production by the fetal lungs and excretion of fetal urine, as well as its intramembranous absorption and swallowing by the fetus. Liquor is formed by diffusion through the skin in the first trimester, whereas in the second and third trimesters by the excretion of hypotonic urine by the fetal kidneys.

Oligohydramnios is the amniotic fluid volume less than the minimum expected for gestational age. Oligohydramnios is observed in nearly 7-8% of pregnancies [1]. Oligohydramnios is caused by factors like placental insufficiency and may lead to fetal growth restriction (FGR) in utero [2]. FGR results in the inability to attain the full genetic potential in terms of growth and development of the fetus and can be due to several fetal, maternal, external, or placental factors. FGR affects nearly 5-10% of pregnancies and is one of the leading causes of perinatal morbidity and mortality [3]. Pregnancies complicated by FGR are increasingly associated with fetal distress or meconium-stained liquor and may also lead to poor birth outcomes like stillbirth, neonatal complications, and higher neonatal intensive care unit (NICU) admissions. FGR is invariably accompanied by impaired uteroplacental blood flow, which results from the failure of or inadequate trophoblastic invasion of the spiral arteries. This leads to insufficient blood flow to the intervillous space [4]. Amino acid supplementation, both orally and in the form of an intravenous infusion, is often prescribed for treating oligohydramnios. Amino acids can adequately provide for the carbon and nitrogen requirements of the growing fetus [5].

L-Arginine is of utmost significance for achieving somatic growth by releasing growth hormone through stimulation of the growth hormone-releasing hormone and the subsequent rise in plasmatic growth hormone [6]. Oligohydramnios associated with FGR is a leading cause of increased operative delivery, perinatal morbidity, and mortality. Most oral and IV interventions are proven to be effective, but any comprehensive study on one modality's guaranteed effectiveness and dosage has yet to be implemented. Therefore, the present study was planned to assess the effect of maternal supplementation with oral vs. intravenous amino acids in cases of oligohydramnios and FGR and its impact on amniotic fluid index and perinatal outcome.

## Materials and methods

Study population

This semi-experimental hospital-based study was done at the Acharya Vinoba Bhave Rural Hospital (AVBRH), Department of Obstetrics and Gynecology, Sawangi, Wardha, for two years, from December 2020 to November 2022, after getting approval from the Institutional Ethics Committee. All participants provided written informed consent. Two study groups, each comprising 52 proven cases of FGR and oligohydramnios by clinical and sonographic assessment in the third trimester attending an antenatal clinic and those admitted to wards, were included in the study.

Inclusion criteria for this study were singleton pregnancy, gestational age between 28-36 weeks, amniotic fluid index (AFI) less than or equal to seven, and FGR grade one on obstetric ultrasonography. Women not willing to give consent to participate, multi-foetal gestation, malpresentation, fetal congenital abnormalities, and premature rupture of membranes were excluded from the study.

Data collection

There were 104 patients fulfilling the inclusion criteria who were admitted for serial monitoring in the ward. Detailed information on the socio-demographic variables like age, address, place of residence, socio-economic status, and their menstrual, obstetric, past, personal, and family history was obtained using a predesigned and pretested proforma. The details about the current pregnancy, such as gestational age and any complications during the present pregnancy, were noted. The patient was advised bed rest in the left lateral position. Intermittent oxygen was supplied. A strict daily fetal movement count was maintained. AFI and estimated fetal weight measurements were done at the time of admission, and the participants were followed on the seventh and 14th day with obstetric ultrasonography after the initiation of treatment.

Ultrasonography was done by a designated sonologist on the same ultrasonography machine to avoid interobserver bias. AFI assessment was done in the supine position using a linear, curvilinear, or sector transducer. While performing the procedure, the uterine cavity is divided into four quadrants. The transducer is placed parallel to the maternal sagittal plane and perpendicular to the coronal plane of the mother throughout the procedure. One of the deepest, free, and unobstructed pockets of amniotic fluid is visualized, and then the image is frozen at that position. Then an ultrasound calliper in a virtual line is placed against the pocket to measure the pocket vertically [7]. The process is similarly performed in the remaining four quadrants, and the pockets are measured vertically and summed to give the AFI. If AFI comes out to be <8, then the procedure is performed in the four-quadrant three times, and the average of the three values is taken [8]. Patients were monitored until delivery. Group A patients received an amino acid IV infusion of 200 ml three times in one week, preceded by the administration of 500 ml of dextrose 5% solution for two consecutive weeks. Group B patients received intravenous (IV) fluid (1 dextrose 10%, 2 Ringer lactate) on an alternate day for two consecutive weeks.

Statistical analysis

The data was analyzed using descriptive and inferential statistics using the chi-square test (x2) and the student's unpaired t-test. The software used in the analysis was IBM SPSS Statistics for Windows, Version 21.0 (Released 2012; IBM Corp., Armonk, New York, United States), OpenEpi Version 3.01, which uses JavaScript and hypertext markup language (HTML) for the calculation of data. A p-value < 0.05 and a 95% confidence interval were considered the level of significance needed to precisely determine the present study's therapeutic effectiveness.

## Results

This study was done at the AVBRH Obstetrics and Gynecology Department, Sawangi Meghe, in Wardha. A total of 104 cases were enrolled for the research after receiving their consent to participate and considering the inclusion and exclusion criteria in pregnancies complicated by oligohydramnios associated with FGR.

Data in Table 1 shows the mean age distribution in the IV amino acid group is 24.11 ± 4.23, and in the IV hydration group is 25.61 ± 4.64 with a p value of 0.511, which is insignificant. The majority of patients were primigravida in both groups. The mean gestational age at admission was 32.73 z± 2.21 and 32.25 ± 2.27, respectively.

**Table 1:** Demographic comparison in IV amino acid infusion and IV hydration group IV: intravenous

Demographic parameters	IV amino acid infusion	IV hydration	P value
Mean age	24.11 ± 4.23	25.61 ± 4.64	0.511
Parity	Primigravida: 39 Multigravida: 13	Primigravida: 37 Multigravida:15	0.69
Mean gestational age at admission	32.73 ± 2.21	32.25 ± 2.27	0.84

As shown in Table 2, in both groups, the mean AFI at admission was observed at 4.93±2.03 cm and 4.22 ± 2.00 cm at the start of therapy. The mean increase in AFI was 1.39 cm and 2.59 cm, respectively, after 7 and 14 days of IV amino acid infusion, whereas 0.83 cm and 1.67 cm after IV hydration therapy.

**Table 2:** Comparison of the effect of IV amino acid and IV hydration on amniotic fluid index in both the groups AFI: amniotic fluid index; IV: intravenous

Amniotic fluid index (in cm)	AFI after IV amino acid	AFI after IV hydration	P value
Mean AFI before intervention	4.93 ±2.03	4.22 ± 2.00	0.91
On the 7^th^ day from the time of admission	6.32± 2.03	5.05 ± 2.09	0.002
On the 14^th^ day from the time of admission	7.52 ±2.04	5.89± 2.20	0.0001

The mean increase in estimated fetal weight (EFW) after seven and 14 days was 149.42 gms and 338.73 gms in IV amino acid group. In the IV hydration group, 95.05 gms and 175.74 gms increase in fetal weight were seen. The mean increase after 14 days was statistically significant compared to seven days as described in Table 3.

**Table 3:** Comparison of estimated fetal weight on USG in IV amino acid and IV hydration group EFW: estimated fetal weight; IV: intravenous

Estimated fetal weight (in gms)	EFW in IV amino acid group	EFW in IV hydration group	P value
At the time of admission	1661.46 ±361.85	1635.06±404.55	0.42
On the 7^th^ day from the time of admission	1810.88 ±367.53	1730.11±400.91	0.53
On the 14^th^ day from the time of admission	2000.19 ±376.70	1810.80±421.05	0.017

**Figure 1:**
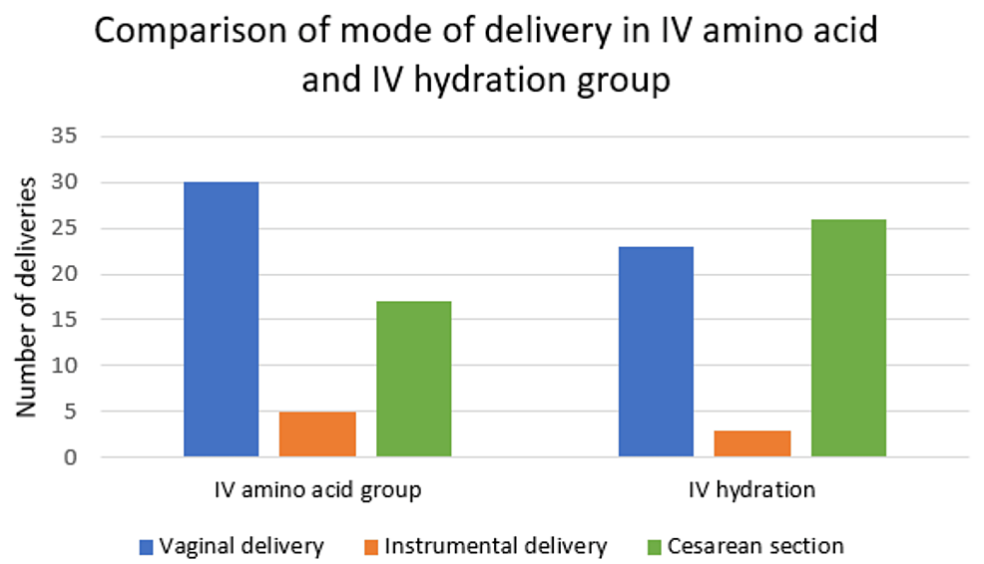
Mode of delivery in IV amino acid and IV hydration group IV: intravenous

The graph in Figure 1 shows a higher number of vaginal deliveries 30 (57.6%) in the IV amino acid group as compared to the IV hydration group 23 (44.23%) with a p value of 0.19 that is statistically not significant. The rate of caesarean section is higher in the IV hydration group (26 out of 52).

Table 4 shows the IV amino acid group had 30 (57.8%) neonates with birth weight of more than 2.5 kg as compared to 18 (34.61%) IV hydration, which is statistically significant. The need for NICU admission was greater in 18 (34.61%) of the IV hydration group than in 11 (21.2%) of the IV amino acid group. One neonatal death was seen in the IV hydration group.

**Table 4:** Neonatal outcome in IV amino acid and IV hydration group IV: intravenous; NICU: neonatal intensive care unit; APGAR: appearance, pulse, grimace, activity, and respiration

Neonatal outcome	IV amino acid group	IV hydration group	P value
Birth weight (> 2.5kg)	30(57.8%)	18(34.61%)	0.018
Mean APGAR at 1 min	6.88 ± 1.16	6.6 ± 1.10	0.288
Mean APGAR at 5 min	8.28 ± 0.93	7.80 ± 1.23	0.022
NICU admission	11(21.2%)	18(34.61%)	0.2644
Neonatal death	0	1(1.92%)	0.31

As shown in Table 5, the most common complications were low birth weight and birth asphyxia, followed by neonatal jaundice, meconium aspiration, hypoglycemia, sepsis, and neonatal deaths. Low birth weight was significantly higher in IV hydration group 34 (65.38%) compared to IV amino acid group 22 (42.30%). 

**Table 5:** Neonatal complications in IV amino acid and IV hydration group IV: intravenous

Neonatal complications	IV amino acid	IV hydration	P value
Low birth weight	22 (42.30%)	34(65.38%)	0.018
Birth asphyxia	10(19.2%)	18(34.7%)	0.031
Meconium aspiration syndrome	9(17.3%)	11(21.2)	0.61
Sepsis	3 (5.7%)	5 (9.6%)	0.46
Hypoglycemia	2(3.8%)	4(7.7%)	0.4

## Discussion

Oligohydramnios causes a significant threat to both the mother and the developing fetus, leading to an increase in perinatal mortality and morbidity. The most important effect is failure to gain effective weight during pregnancy, leading to FGR. Monitoring the amniotic fluid index is vital for early recognition of oligohydramnios associated with FGR to institute timely corrective measures. In this study, the outcomes of 104 patients diagnosed as having oligohydramnios associated with FGR were compared across two groups, the IV amino acid infusion group, and the IV hydration group, and their effect on amniotic fluid index and the perinatal outcome was observed. The observations of this study have been discussed and compared with those of other studies.

In the present study, the majority 39 (75.0%) and 37 (71.2%) of the cases from the IV amino acid and IV hydration groups, respectively, were primigravida. There was no statistical significance in the groups with regard to their parity. Sharma et al. report that 37 out of 50 (74%) cases were primigravida [9]. The higher risk of acquiring oligohydramnios among primigravida may be due to the fact that the underlying disorders of pregnancy are exaggerated in primigravida due to maladjustment of the body to the developing fetus. Soni et al. report primigravida at 74 out of 100 (74%) and multigravida at 26% [10].

Gestational age

The present study showed that the mean gestational age in the IV amino acid group was 32.73 ± 2.21 weeks and in the IV hydration group, 32.25 ± 2.77 weeks, which was statistically insignificant. Other previous studies, like the one by Ropacka et al., revealed a mean gestational age at diagnosis in the study group of 31.2 + 3.1 weeks compared to 29.7 + 3.4 weeks in the control groups [11]. It was also comparable to another study by Dera et al., in which the mean gestational age at admission was 31.09 + 2.98 weeks in the study group and 29.88 + 3.22 weeks in the control group [12]. A study by Mohamed et al. observed a decrease in the amniotic fluid with advancing gestational age, which contradicts the present study [13]. They report a decline in the amniotic fluid with increasing gestational age.

AFI

The initial AFI scores in the IV amino acid and IV hydration groups at the time of admission were comparable in both groups (p = 0.91; NS). Azarkish et al. had 4.49 ± 0.34 cm AFI when administering IV amino acids [14]. Qureshi FU et al. had a similar mean AFI at the time of pre-infusion, 4.7 ± 1.58 cm [15]. The mean AFI at the time of admission, on the seventh and 14th days, was comparatively higher in the IV amino acid group. These findings were similar to those obtained by Bhargava et al., who reported a significant improvement in the amniotic fluid volume with the intravenous amino acid at the end of 14 days of follow-up. In their study, at the end of two weeks, there was a reduction in the patients with AFI < 5 from 20 to just 15, and at the end of four weeks, only 12 patients with AFI ≤ 5 cm were present. Similarly, patients with AFI between 5.1-10 cm also improved from 40 to 18 at the end of four weeks. Out of 60 patients that were enrolled, 50% showed an improvement in the AFI after four weeks of treatment with IV amino acids [16]. Amino acids can adequately provide for the carbon and nitrogen requirements of the growing fetus. They also regulate the development of the fetus and placenta. They serve as the protein building blocks for fetal growth and the development of the metabolic cyclic pathways between the placenta and fetus. Supplementation of amino acids improves vascularity by enhancing nitric oxide synthesis and stimulating insulin secretion, which is a major contributor to appropriate fetal growth. A significantly higher increase in AFI was seen in the IV amino acid group as compared to the IV hydration group. A comparison of IV amino acid infusion with various other studies is described in Table 6.

**Table 6:** Comparison of results of AFI in IV amino acid infusion group with previous IV amino acid infusion studies AFI: amniotic fluid index; IV: intravenous

Studies	Pre treatment AFI	Post treatment AFI	Mean increase AFI
Present study	4.93 ±2.03	7.52 ±2.04	2.59
Habib et al., 2021 [17]	3.73 ± 0.36	6.82 ± 0.62	3.09 ± 0.70
Sunil I et al., 2019 [18]	5.1 ± 1.35	7.64 ± 0.67	2.32 ± 0.67
Shree P et al., 2016 [19]	5.56 ± 2.3	8.13 ± 2.98	2.57 ± 0.68
Qureshi et al., 2011 [15]	4.7 ± 1.58	6.2 ± 1.75	1.5 ± 0.17

EFW

When the EFW was compared in both groups, it was observed that the mean EFW at the time of admission was similar in both groups. Habib et al. found a 3.09 cm increase in AFI post-transfusion [17]. In Sunil et al.'s study, pretreatment AFI was 5.1 ± 1.35 cm and a 2.32 ± 0.67 of increase in AFI was seen post-transfusion [18]. The average gain in fetal weight in the IV amino acid group was 150 ± 37.8 gms than in the control group p < 0.01, which is statistically significant in Shree P et al. at the end of one week [19]. According to Shivkumar PV et al., in groups that received amino acids, the weight gain of the fetus was much greater than in groups that received no treatment or only intravenous fluids. Maximum fetal weight gain was in amino acid infusion and five pints of hydration of normal saline, ringer lactate, and 5% dextrose, i.e., 40% between 400 to 500 gms [20]. However, in a study conducted by Rinehart et al., the results were contradictory to the ones obtained in the present study, as they showed insignificant weight gain with amino acid supplementation [21]. This variability in the EFW gain patterns observed across the studies might be attributable to their varying inclusion criteria, as some of them enrolled the women in the second trimester, and depends on the duration of treatment that varies in different studies.

Mode of delivery

A higher number of vaginal deliveries was seen in IV amino acid group 30 (57.6%). Sharma et al. report that caesarean delivery is 35 out of 50, which is 70% [9]. The rate of cesarean delivery noted by Soni et al. is 72%, with the main indication of fetal distress (46%) [10]. Shivkumar et al. report more normal IV amino acid infusion deliveries without complications [20]. Hundred out of 82 were delivered vaginally, with ten instrumental deliveries and eight caesarian sections. One of the studies by Morris et al. reported a higher rate of caesarean section (LSCS) that was mainly due to fetal distress as a result of cord compression and variable deceleration [22]. They have also reported that the increased LSCS rate in cases with oligohydramnios might be due to meconium-stained liquor, non-reactive cardiotocography (CTG) due to reduced amniotic fluid volume, and FGR.

Neonatal complications

IV hydration group 18 (34.61%) has more NICU admissions than IV amino acid group 11 (21.32%). One neonatal death was seen in the IV hydration group. The major underlying factor is uteroplacental insufficiency and the resulting umbilical cord compression. These may even lead to fetal demise in utero. Hence, these are strong indications for the termination of pregnancy with oligohydramnios [23]. Shivkumar PV et al. reports one neonatal death due to severe birth asphyxia with extremely low birth weight in the IV amino acid group compared to three neonatal deaths in the control group [20]. The study by Bhargava et al. also reported that adverse neonatal events were significantly lower in the IV amino acid group compared to the control [16]. The incidence of fetal complications during labor, like meconium staining of liquor, was considerably lower in the IV amino acid group than in the control group.

Limitations

Our findings might not be generally applicable beyond the study group, which includes pregnant women in similar rural and urban environments, because the study was conducted in a single centre at a tertiary care facility with a smaller sample size.

## Conclusions

The present study shows improvement in AFI, EFW, and perinatal outcomes on IV amino acid and IV hydration therapy. The intravenous amino acid infusion is an effective modality for treating oligohydramnios associated with FGR, with a strong positive impact on the amniotic fluid index after seven and 14 days of supplementation. The rate of vaginal delivery, increased baby weight at birth with a higher appearance, pulse, grimace, activity, and respiration (APGAR) score at one minute and five minutes, as well as lesser neonatal complications, were seen in the IV amino acid infusion group.
